# Impact of stomatitis on pain relief and nutrition in palliative radiotherapy using quad shot: a prospective study

**DOI:** 10.1093/jrr/rraf058

**Published:** 2025-09-23

**Authors:** Osamu Tanaka, Kosuke Naganawa, Takashi Matsuzuka, Yuichi Ehara, Yasuhisa Hasegawa, Takuji Kiryu, Akira Ukai, Chiyoko Makita, Masayuki Matsuo

**Affiliations:** Department of Radiation Oncology, Asahi University Hospital, 3-23 Hashimoto-cho, Gifu City, Gifu 500-8523, Japan; Department of Oral and Maxillofacial Surgery, Asahi University Hospital, 3-23 Hashimoto-cho, Gifu City, Gifu 500-8523, Japan; Department of Head and Neck Surgery, Asahi University Hospital, 3-23 Hashimoto-cho, Gifu City, Gifu 500-8523, Japan; Department of Oral and Maxillofacial Surgery, Asahi University Hospital, 3-23 Hashimoto-cho, Gifu City, Gifu 500-8523, Japan; Department of Head and Neck Surgery, Asahi University Hospital, 3-23 Hashimoto-cho, Gifu City, Gifu 500-8523, Japan; Department of Radiology, Asahi University Hospital, 3-23 Hashimoto-cho, Gifu City, Gifu 500-8523, Japan; Department of Oral and Maxillofacial Surgery, Asahi University Hospital, 3-23 Hashimoto-cho, Gifu City, Gifu 500-8523, Japan; Department of Radiology, Gifu University Hospital, 1-1 Yanagido, Gifu City, Gifu 500-1141, Japan; Department of Radiology, Gifu University Hospital, 1-1 Yanagido, Gifu City, Gifu 500-1141, Japan

**Keywords:** head and neck cancer, pain, quality of life, radiotherapy, stomatitis

## Abstract

Quad Shot (QS) is effective in treating head and neck cancer; however, few prospective studies have been conducted in this direction. Further, no studies have investigated tumor pain and stomatitis pain separately. We prospectively investigated the efficacy and adverse events of QS in 11 patients with head and neck cancer who underwent QS at our hospital in Japan between 2018 and 2024. The QS method involved administering 3.7 Gy twice daily for 2 days, which was considered one course and provided thrice at an interval of 4 weeks. We assessed quality of life (QOL) scores, albumin levels, and numerical rating scale (NRS) scores for stomatitis and tumor pain before and after QS to evaluate changes in NRS. Eleven patients with advanced head and neck cancer received QS treatment: six patients underwent three courses, three underwent two, and two underwent one. There was no significant difference in QOL scores before and after QS, but albumin levels dropped significantly after QS. NRS due to stomatitis significantly worsened after QS, whereas NRS due to tumor significantly improved. Tumor size decreased and tumor NRS improved as the QS treatment duration increased. However, stomatitis was almost always present, and NRS scores for stomatitis increased significantly after treatment. In conclusion, QS can alleviate tumor pain but may worsen stomatitis. Therefore, stomatitis care should be emphasized during treatment. Furthermore, the decrease in albumin levels is likely due to stomatitis-induced decreased appetite; therefore, stomatitis management is also important for maintaining nutritional status.

## INTRODUCTION

Definitive irradiation is not recommended for patients with head and neck cancer who are in poor general health, including those unable to tolerate prolonged treatment or adverse events associated with radiation therapy (RT) or combined chemoradiotherapy, those with highly advanced local disease for whom definitive irradiation is difficult, or those with distant metastasis. However, even in such cases, palliative RT is considered effective if the patient has symptoms such as difficulty swallowing, hoarseness, pain, or bleeding [1–3].

Among these hypofractionated radiotherapy methods, Quad Shot (QS), which consists of four shots over 2 days in one course, has been shown to be an effective treatment for head and neck cancer in patients with significantly reduced quality of life (QOL) due to pain, bleeding, difficulty swallowing, and cosmetic problems, primarily in studies from Europe and the United States [2–11].

Because setting endpoints in retrospective studies is difficult, prospective studies are necessary to demonstrate clinical significance. In Japan, several studies have evaluated the clinical utility of the QS regimen for the palliative treatment of head and neck cancer. Toya *et al*. [5] retrospectively reported symptom control and tumor response using volumetric modulated arc therapy–based QS. Additionally, Toya *et al*. [12] recently conducted a prospective study on QOL improvement and tolerability. Most previous studies have assessed oral pain using a single method, such as a numerical rating scale (NRS); however, in clinical practice, stomatitis in the irradiated area is a well-recognized issue among patients. We designed the present study with close reference to these previous studies, introducing a novel element: the clinical differentiation and analysis of tumor-related pain and stomatitis-related pain, which remains underreported in palliative care.

## MATERIALS AND METHODS

The analysis involved 11 patients with head and neck cancer who underwent QS at our hospital between 2018 and 2024. This prospective observational study was approved by the Institutional Review Board of Asahi University Hospital (approval number 2018-09-01). All participants provided written informed consent before enrollment. This study was conducted in accordance with the principles of the Declaration of Helsinki.

The inclusion criteria for this study were established in alignment with previous Japanese studies by Toya *et al*., which evaluated QS radiotherapy for incurable head and neck cancer [10, 12]. Specifically, eligible patients were aged ≥18 years, had histologically confirmed head and neck cancer with a measurable tumor in the irradiated area, and were considered unsuitable for curative treatment. Although our inclusion criteria allowed patients with an Eastern Cooperative Oncology Group (ECOG) performance status (PS) ≤4, only patients with PS 1 or 2 were finally enrolled, reflecting real-world treatment feasibility. Exclusion criteria included prior radiotherapy to the same region, concurrent systemic chemotherapy, and the presence of uncontrolled systemic disease.

Given the rarity of QS indication in head and neck cancer and its palliative context, the sample size was determined pragmatically. This pilot analysis aimed to generate preliminary data and explore the feasibility and patterns of pain differentiation rather than confirm statistical hypotheses.

**Fig. 1:**
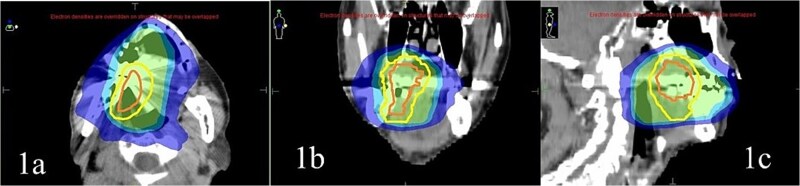
Irradiation area; (a) axial view, (b) coronal view, (c) sagittal view. Gross target volume (GTV) (orange line): Region of visible lesion and tumor extension identified by FDG-PET, contrast-enhanced CT, and magnetic resonance imaging. Clinical target volume (CTV): Defined by adding a 5-mm margin to the GTV. PTV (Yellow line): Defined by adding a 5–10 mm margin to the CTV. Regions receiving 95%, 80%, and 50% of the prescribed dose are indicated in light green, light blue, and blue, respectively. GTV, Gross target volume; CTV, Clinical target volume; PTV, Planning target volume; FDG-PET, Fluorodeoxyglucose positron emission tomography.

The QS method involved administering 3.7 Gy twice a day (9:00 and 15:00) for 2 days (a total of 14.8 Gy). This was scheduled as a single course, with three courses being provided at 4-week intervals. The irradiation site was the planning target volume (PTV). The PTV was determined by adding a 1-cm margin to the gross target volume (GTV). The GTV was identifiable through imaging. Table 1 describes the patient characteristics. Figure 1 shows a sample of radiotherapy planning images.

**Table 1:** Characteristics of the patients (*n* = 11)

Age (years)		Median 81 (range: 54–92)
Gender		
Male		9
Female		2
ECOG performance status		
0		4
1		7
2		0
3		0
Tumor site		
Oral cavity		7
Nasal cavity and paranasal sinuses		4
Histology		
Squamous cell carcinoma		11
Others		0
Clinical T stage		
1		1
2		2
3		7
4		1
Opioid		
Yes		3
No		8
Bleeding		
Yes		6
No		5
QOL before RT		24.3 ± 7.2
Alb before RT		3.5 ± 0.3
NRS: Stomatitis before RT		0.1 ± 0.3
NRS: tumor pain before RT		3.3 ± 2.4
GTV size at first QS	11	279 ± 259.7
GTV size at second QS	9	284.3 ± 270.6
GTV size at third QS	6	167.1 ± 137.6

Abbreviations: QS, Quad Shot; QOL, Quality of life; Alb, Albumin; GTV, Gross target volume (cc); NRS, Numerical rating scale; RT, Radiotherapy.

### Assessment of the timing

The primary endpoint was the change in pain intensity as measured by the NRS, making it easy to understand objectively. The primary causes of oral pain were tumors and stomatitis, and their severity was measured separately using the NRS. QOL scores, albumin levels, and NRS (stomatitis and tumor) scores were assessed before (within 3 days before the first QS course) and at follow-up (4 weeks after each QS course). NRS was reported before and after QS when the value was highest. If the pain worsened during the treatment period, analgesics were administered and the NRS score was recorded.

Symptomatic treatment for mucositis pain was provided using a mouthwash solution containing lidocaine and azulene sulfonate sodium in accordance with the Multinational Association of Supportive Care in Cancer/International Society of Oral Oncology (MASCC/ISOO) guidelines [13]. Patients were instructed to hold the solution in their mouth for 30 s to 1 min and spit it out. The mouthwash was administered approximately 10–15 min before meals, as the local anesthetic effect of lidocaine typically lasts for 15–30 min. Thorough instructions were provided to prevent complications such as aspiration, and no adverse events were observed. The mouthwash composition did not include systemic analgesics or corticosteroids. The nursing staff supervised the administration during hospitalization, and the outpatients were provided with instruction sheets. Pain relief from using the mouthwash was not measured in the NRS.

### Quality of life analysis

European Organization for Research and Treatment of Cancer Quality of Life Questionnaire Core 30 (EORTC QLQ-C30): An objective of palliative care for patients with cancer is improving their QOL. There are numerous general and disease-specific QOL assessments for patients with cancer; however, the EORTC QLQ-C30 is widely used. The EORTC QLQ-C30 was developed in Europe and has been translated into 81 languages. Its reliability and validity have been confirmed. The scale encompasses multiple functional and symptom domains; however, only the global QOL score was retained in digital format because of ethical concerns about including patient identifiers on paper-based forms. Therefore, domain-specific analysis was not feasible in this study. Nevertheless, improvement in the global QOL score indicates an overall enhancement in patient well-being after QS radiotherapy.

All analyses were performed using BellCurve for Excel® (Social Survey Research Information Co., Ltd., Tokyo, Japan), with significance defined as *P* < 0.05. No missing data were observed.

## RESULTS

**Fig. 2:**
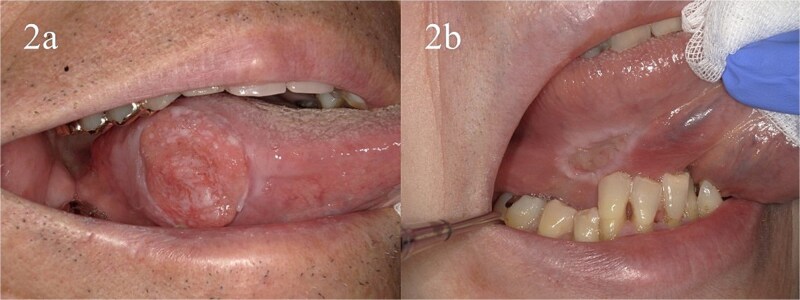
Presentation of a 91-year-old man with pain in the tongue. Macroscopic finding: At the initial consultation, the tongue tumor measured 30 and 25 mm along its major and minor axes, respectively. The depth of invasion (DOI) was 10 mm, and there was no lymph node metastasis; thus, it was diagnosed as T3N0M0 stage 3 (a). The patient refused surgery and underwent two courses of Quad Shot (QS) as palliative treatment. The tumor size was significantly reduced using QS (major axis: 20 mm; minor axis: 15 mm; DOI: 7 mm) (b). Subsequently, the patient requested surgery and underwent partial tongue resection. The tumor measured 16 and 13 mm along the major and minor axes, respectively. The DOI was 7 mm, and no signs of recurrence were observed for 18 months after treatment. Following QS, the NRS for the tumor decreased from 6 to 0, whereas the NRS for stomatitis increased from 0 to 3. Stomatitis resolved within 2 weeks. No other adverse events were observed.

**Fig. 3:**
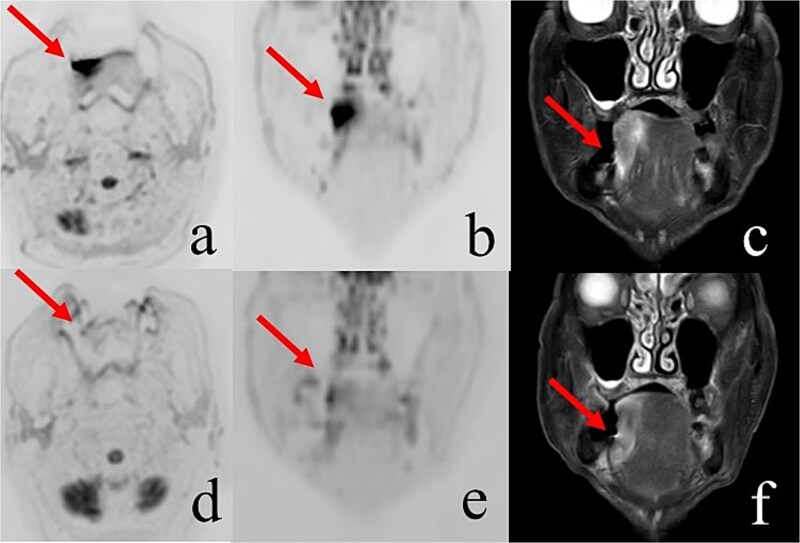
Magnetic resonance imaging findings before and after treatment*.* The images in the top row (a, b, and c) are from before the Quad Shot, and the images in the bottom row (d, e, and f) are from 2 months after the Quad Shot. Diffusion-weighted images show abnormal signals before treatment (a and b); however, these signals disappeared 2 months after treatment (d and e). No changes are visible in the short tau inversion recovery (STIR) images (c and f), as diffusion-weighted imaging reflects changes in the internal structure of the lesion earlier than STIR.

Six patients received three QS treatments, three received two, and two received one. Figures 2 and 3 depict oral tumor progression in a patient who completed two courses of treatment. Six patients who experienced bleeding were successfully treated via QS treatment. The remaining two patients experienced slight bleeding even after QS treatment; however, further treatment was not needed, as the tumor-related bleeding was not sufficiently severe. Stomatitis necessitated an increase in analgesic use in 4 of the 11 patients.

No significant difference was observed in QOL before and after QS (Table 2). However, albumin levels decreased significantly after QS. Furthermore, NRS due to stomatitis significantly worsened following QS, whereas NRS due to tumors improved significantly.

**Table 2:** Changes in values before and after RT

		*P*-value
QOL score		0.793
Before RT	24.3 ± 7.2	
After RT	24.0 ± 7.0	
Albumin level		0.001
Before RT	3.5 ± 0.3	
After RT	3.0 ± 0.3	
NRS of Stomatitis		0.005
Before RT	0.1 ± 0.3	
After RT	2.7 ± 2.6	
NRS of the tumor		0.033
Before RT	3.3 ± 2.4	
After RT	2.4 ± 2.4	

The patients’ condition was subjectively assessed before and after radiotherapy.

Paired t-test was used to compare before and after treatment values, with a significance level of *P* < 0.05.

QOL did not change during the treatment period, but albumin levels decreased significantly. Tumor pain decreased, but stomatitis pain increased.

Abbreviations: QOL, Quality of life; NRS, Numerical rating scale; RT, Radiotherapy.

NRS deterioration due to stomatitis and that due to tumor were compared using univariate analysis. We examined ECOG PS, tumor site, histology, clinical tumor stage, opioid use, bleeding, initial QOL, albumin, and gross tumor volume. None of these variables significantly affected NRS.

Three patients could only receive two courses of QS treatment because of deterioration in their overall health, which prevented them from visiting the hospital. One patient received only one QS treatment because he refused additional treatment due to stomatitis. Another patient has been admitted to a home medical facility.

As the number of QS treatments increased, tumor size decreased and tumor NRS improved; however, stomatitis was almost always present and was statistically significantly more severe than before QS treatment.

## DISCUSSION

### Current treatment standard for advanced head and neck cancer

An important goal of cancer treatment is to help patients lead normal lives and prevent QOL decline until the end of life. Even in the late disease stages, patients and their families should be supported in maintaining their individuality and living a comfortable life; thus, ‘best supportive care’ is critical for patients with terminal cancer. Local tumor control is especially important in patients with head and neck cancer, including oral cancer, because maintaining tissue form and function significantly affects their QOL.

In head and neck cancer with no distant metastases, the end of life is marked by local tumor growth; patients witness the tumor enlarging while experiencing worsening functional impairments and bleeding, which increases mental burden. Furthermore, because of limitations in managing bleeding and necrotic tissue in the oral cavity, face, and neck, controlling tumor growth and associated symptoms is particularly crucial in these patients compared with those with other cancer types.

When radical treatment becomes difficult for patients with advanced cancer, the treatment shifts to palliative care aimed at local control, with palliative RT being one such approach. In recent years, palliative RT has emerged as the gold standard for treating brain metastasis, bone metastasis, superior vena cava syndrome, and spinal cord compression, leading to improved QOL and reduced symptoms.

### Methods of palliative radiotherapy

Palliative RT for head and neck cancer can be performed using various dose fractionation schedules, with the most popular being 30 Gy/10 and 8 Gy/1 fractions. Hypofractionated radiation therapy is may decrease tumor size and alleviate symptoms early by increasing the dose per fraction and decreasing the total dose. However, 30 Gy/10 fraction hypofractionated RT is deemed inappropriate for head and neck cancer because of its high rate of grade 3 or higher acute adverse events (≥40%). Other hypofractionated RT techniques also have good symptom relief rates; however, the incidence of acute adverse events is high, and more effective palliative treatment methods are needed when considering QOL.

### Quad shot

Each course of QS, a form of hypofractionated radiation therapy, comprises four doses. This method delivers approximately twice the amount of radiation per dose compared with conventional fractionation (1.8–2.0 Gy), with doses administered in the morning and evening over 2 days, totaling 14.8 Gy per course. Three to four doses of each treatment are given at 3–6-week intervals [2–11, 14, 15]. Compared with standard radiation therapy, QS is characterized by rapid tumor shrinkage and the potential for early symptom relief.

This treatment provides effective symptom relief within a short period while minimizing the risk of acute adverse events. For instance, increasing the radiation dose per session can reduce the total number of irradiation sessions, easing the burden on the patient. QS is also effective as a palliative treatment for the rapid improvement of localized symptoms. Many clinical studies have found that QS promotes early tumor shrinkage and improves patient QOL [2–11, 14, 15].

However, the number of acute adverse events caused by QS remains high. The frequency of adverse events is concerning, particularly in patients with head and neck cancer. Acute mucosal damage, bleeding, and increased pain have been reported, often necessitating additional treatment. Therefore, treatment plan should consider tumor location and progression as well as the patient’s overall health and QOL. A key finding of this study is the consistent occurrence of mucositis-related pain during QS treatment, as evidenced by the significant increase in stomatitis NRS scores. Unlike tumor pain, which can often be managed with nonsteroidal anti-inflammatory drugs (NSAIDs) and opioids, mucosal damage pain remains difficult to control pharmacologically.

Clinically, many patients initially experience tumor-localized pain that improves after QS; however, mucosal pain subsequently emerges as the predominant symptom, particularly during the peak phase of mucositis 1–2 weeks post-irradiation. This shift in pain origin presents a unique challenge in palliative care and underscores the need for supportive interventions.

The use of analgesics may have influenced the NRS scores. In clinical trials involving pain from a single source, a ≥ 25% reduction in analgesic use with no worsening in NRS may indicate a therapeutic effect. However, the primary goal in the palliative care setting is complete pain relief (i.e. NRS = 0), not reduced medication use. Therefore, analgesic doses are adjusted to the maximum tolerated levels based on each patient’s condition, and fixing or reducing analgesics solely for study purposes is ethically and clinically inappropriate. Although analgesic use was documented in this study, it was not standardized across patients.

### Differences in pain between tumors and stomatitis after quad shot

We considered both tumor-related pain and stomatitis-associated pain after QS. Pain is measured using the NRS. Stomatitis often reduces food intake, lowering albumin levels and further increasing the risk of stomatitis, thus creating a vicious cycle. Therefore, early adjuvant therapy for stomatitis is necessary during QS treatment.

As a preventive measure, we performed gastrostomies for patients who requested them. Patients were instructed to hold 100 ml of xylocaine-containing water in the mouth before meals to induce mild oral numbness and alleviate eating-related pain. Although gastrostomy can be difficult for terminally ill patients, it is effective in maintaining albumin levels. We also believe that the xylocaine-containing mouthwash was beneficial. Our findings show that stomatitis pain can be as severe as tumor pain; therefore, early treatment is critical.

Although mucositis and a transient drop in albumin levels are common during QS course, these effects are typically manageable and temporary. Moreover, when the scheduled treatment is completed, patients often experience significant relief from tumor-related symptoms, including bleeding and pain, leading to a net improvement in overall QOL.

The QS regimen involves only two consecutive days of treatment, repeated every 4 weeks, which is substantially less burdensome than conventional daily RT over several weeks. For patients with limited life expectancy or impaired performance status, this condensed schedule represents a patient-centered balance between treatment efficacy and preservation of daily living.

### Relationship between albumin and stomatitis

Reduced oral intake typically leads to nutritional decline. In cases of stomatitis, pain throughout the mouth makes normal eating difficult, necessitating the use of blended foods and nutritional supplements to facilitate intake. This makes it difficult to obtain adequate nutrition, and the albumin levels drop. Stomatitis is more likely to occur if albumin levels drop, creating a vicious cycle. Therefore, stomatitis management should be conducted in accordance with the MASCC/ISOO guidelines [13].

### Literature analysis

Palliative radiation therapy for advanced head and neck cancer has been studied extensively in Europe and the United States. Grewal *et al*. found that administering QS as palliative radiation therapy for advanced head and neck cancer reduces adverse events and prioritizes patient comfort. They found that QS can improve QOL during treatment and is a viable option for patients [4].

Toya *et al*. [5] demonstrated that QS can cause tumor shrinkage or symptom relief in approximately 80% of patients with few adverse events. Another report from Toya *et al*. [12] concluded that only 2% of patients (*n* = 2) developed grade 3 toxicity. Our study extends these findings by specifically differentiating tumor-related pain from stomatitis-related pain, an important but previously underexplored aspect in the context of QS. Furthermore, multiple irradiation courses are recommended for this treatment, indicating a significant impact on the ongoing treatment. Therefore, this study demonstrates that QS is extremely effective for patients with advanced cancer and helps maintain QOL during treatment.

Choudhary *et al*. [6] compared the conventional hypofractionated radiation method (30 Gy/10 fractions/2 weeks) with QS (14.8 Gy/4 fractions/2 days). Although there was no significant difference in symptom relief (pain, dysphagia, and hoarseness), QS significantly reduced adverse events such as radiation dermatitis and stomatitis, indicating that it has palliative effects while suppressing adverse events.

Corry *et al*. [3] administered QS treatment shortly before the onset of oral mucositis. The treatment protocol included a several-week interval between each course, allowing for mucosal stem cell recovery and contributing to a reduction in adverse events. Thus, QS is a suitable treatment method for head and neck cancer, minimizing adverse events.

Fan *et al*. [7] found that 86% of patients with advanced head and neck cancer who received three or more QS courses experienced palliative effects and increased survival time. This demonstrates that QS is effective in patients with cancer and does not impede cancer treatment.

### Limitations

In this study, analgesics were administered at the maximum tolerable dose throughout the treatment consistent with standard palliative care. Tumor-related pain was generally well-controlled with NSAIDs and opioids, whereas mucositis-related pain was less responsive to these medications. Notably, patients often reported a shift from focal tumor pain to diffuse oral discomfort—suggesting a qualitative change in pain, not simply a reduction. Although analgesic use was documented, it was not standardized across patients. Future studies should consider quantifying analgesic consumption using morphine equivalent daily dose to better control for this variable.

In conclusion, there are no universal guidelines for palliative care for patients with advanced head and neck cancer; therefore, selecting a treatment method that is specific to each patient is crucial. QS is a palliative radiotherapy that provides good symptom relief and local response while preserving and improving QOL with few side effects. Treatment can be provided in a short-term hospital setting and for outpatients. The crucial aspect of these findings, which has not been previously investigated, is the separate consideration of tumor pain and stomatitis pain. We believe that early intervention for stomatitis pain will increase QS course completion rates and that developing a nutritional strategy at the outset of treatment is critical.

## Data Availability

All data will be made available upon responsible request by the corresponding author.
